# Pregnancy in Thalassemia and Sickle Cell Disease: The Experience of an Italian Thalassemia Center

**DOI:** 10.3389/fmolb.2020.00016

**Published:** 2020-02-14

**Authors:** Francesco Sorrentino, Laura Maffei, Patrizia Caprari, Rita Cassetta, Donatella Dell'Anna, Stefano Materazzi, Roberta Risoluti

**Affiliations:** ^1^Thalassemia Unit, S. Eugenio Hospital, Rome, Italy; ^2^National Centre for the Control and Evaluation of Medicines, Istituto Superiore di Sanità, Rome, Italy; ^3^Gynecology Unit, S. Eugenio Hospital, Rome, Italy; ^4^Immunohematology and Transfusion Medicine, S. Eugenio Hospital, Rome, Italy; ^5^Department of Chemistry, Sapienza University of Rome, Rome, Italy

**Keywords:** pregnancy, thalassemia major, thalassemia intermedia, sickle cell disease, pregnancy management

## Abstract

The life expectancy of thalassemia patients has increased significantly in recent years being the most “elderly” patients approaching or are over 50 years old. Consequently, patients' perspectives have changed, leading them to longer-term planning with a consequent increase in their reproductive potential and desire to have children. Crucial points in the management of pregnancy in thalassemia are the iron chelation therapy before and during pregnancy, the antithrombotic prophylaxis, the management of transfusion therapy according to the modified transfusion requirement, a cardiologic monitoring for hemodynamic changes that expose an increased risk of heart failure. Pregnancy in women with sickle cell disease is still associated with increased rates of maternal and fetal mortality and adverse outcomes. Maternal morbidity may be due to acute sickling crises, thromboembolism, infection, and chronic end-organ dysfunction, while neonatal outcomes may be intrauterine growth retardation, preterm delivery, small infants for gestational age, stillbirth, and neonatal death. The management of pregnancy in thalassemia and sickle cell disease requires to be approached by a multidisciplinary team and followed from the pre-conception phase until the post-partum period with a close monitoring of the maternal and fetal conditions, in order to ensure optimal outcome. This approach requires the application of well-defined protocols that cover all the critical aspects of pregnancies in women affected by these pathologies. We describe our experience of spontaneous and non-spontaneous pregnancies in patients with thalassemia major and intermedia and sickle cell disease followed between 1992 and 2018 at the Thalassemia Unit of S. Eugenio Hospital of Rome.

## Introduction

The natural history of thalassemia has drastically changed in recent years thanks to the improvement of transfusion and iron chelation therapy and to the treatment of complications derived from them. The patients' life expectancy has increased significantly, being the most “elderly” patients approaching or are over 50 years old. Younger patients are those who benefit most from improved therapy, presenting fewer complications related to martial overload. The increase in life expectancy has consequently changed patients' perspectives, leading them to longer-term planning with a consequent increase in their reproductive potential and desire to have children. Hypogonadism still represents an obstacle for some patients, but the progress in assisted reproduction techniques has helped many of them to fulfill this wish (Leung and Lao, 2012; Lao, 2017; Materazzi et al., 2017a; Carlberg et al., 2018; Origa and Comitini, 2019).

Pregnancies in women with sickle cell disease (SCD) are associated with increased rates of maternal and fetal mortality and adverse outcomes (Eissa et al., 2015; Kuo and Caughey, 2016; Ware et al., 2017; Jain et al., 2019; Patel et al., 2019). The physiological changes of pregnancy (increased metabolic demand, rise of blood viscosity, hypercoagulability) are aggravated in SCD women leading to increased incidence of complications. Maternal morbidity may be due to acute sickling crises, thromboembolism, infection and chronic end-organ dysfunction, while neonatal outcomes may be intrauterine growth retardation, preterm delivery, small infants for gestational age, stillbirth, and neonatal death. Early reports on the outcome of pregnancy in SCD depicted an almost universal adverse outcome for mother and child, but the medical care improvements, like the introduction of pre-conceptional care, have reduced the risk of complications.

In this retrospective study, we report the experience of pregnancies in patients with thalassemia major and intermedia and sickle cell disease followed at the Thalassemia Unit of S. Eugenio Hospital of Rome. Moreover we report the maternal and fetal outcomes of the patients comparing with the experiences previously reported in literature (Tuck, 2005; Origa et al., 2010; Leung and Lao, 2012; Voskaridou et al., 2014; Lao, 2017; Carlberg et al., 2018; Jain et al., 2019).

## Methods

We included in the study pregnant women with diagnosis of Thalassemia and SCA obtained through a comprehensive assessment of clinical presentation, screening tests, and molecular characterization of globin genes mutations (Greene et al., 2014; Aiello et al., 2015; Risoluti et al., 2016, 2018; Materazzi et al., 2017b,c; Catauro et al., 2018). The study was approved by Comitato Etico of the S. Eugenio Hospital and all the patients provided their written informed consent to participate in this study.

We have followed between 1992 and 2018 the course and outcome of spontaneous and non-spontaneous pregnancies from 31 women, 21 of them were patients of the Thalassemia Unit of S. Eugenio Hospital of Rome, while 10 were patients from other hospitals and were followed at the Thalassemia Unit during the pregnancy for the complications.

The evaluation was performed by analyzing the data of all the patients and also divided in to the following groups: thalassemia major, thalassemia intermedia transfusion-dependent, thalassemia intermedia non-transfusion dependent, and sickle cell disease.

The following parameters were analyzed:

1. Clinical and demographic characteristics: age of the patients at the pregnancy, country of origin, transfusion frequency, hypogonadism, splenectomy, iron chelation therapy.
2. Pregnancy: conception, fertilization *in vitro*, intrauterine insemination after induced ovulation.
3. Treatment during pregnancy: transfusion, iron chelation, complications.
4. Delivery: gestational age at delivery, preterm delivery, abortion, cesarean delivery.
5. Neonatal outcomes: birth weight, fetal anomalies, complications.

## Results

### Clinical and Demographic Characteristics

The course and outcome of 33 pregnancies from 31 patients, 25 thalassemic patients and 6 women with SCD have been analyzed retrospectively, and the characteristics of the patients are described in Table 1.

**Table 1:** Pregnancy in thalassemia and sickle cell disease: the experience of an Italian Thalassemia Center.

**Patients**		**All (*n* = 31)**	**TM (*n* = 14)**	**TD-TI (*n* = 7)**	**NTD-TI (*n* = 4)**	**SCD (*n* = 6)**
Age of patient at the pregnancy, years	Mean	38.9	33.2	34.0	34.5	32.1
	Range	25–46	25–46	25–40	29–42	25–43
Origin, *n* (%, country)		27 (87%, Italy) 3 (10%, Africa) 1 (3%, Albania)	14/Italy	7/Italy	4/Italy	2/Italy 3/Africa 1/Albania
Regular transfusion, *n* (%)		23 (74%)	14	7		2
Hypogonadism, *n* (%)		8 (26%)	7	1		
Splenectomy, *n* (%)		16 (52%)	11	4		1
Pregnancies, *n* (%)	All	33	14	8	5	6
	Spontaneous	21 (64%)	7	3	5	6
	Induced	12 (36%)	7	5		
Twin pregnancy, *n* (%)		3 (9%)	2	1		
Iron chelation (DFO) (2nd−3rd trimester of pregnancy), *n* (%)		14 (42%)	10	3		1
Complications of patients, *n* (%)	Heart failure	1 (3%)	1			
	VOC	2 (6%)				2
Mean gestational age at delivery (weeks)		37.1 ± 2.2*	36.3	37.1	38.4	37.3
Preterm delivery, *n* (%)		7 (22%)	5	1		1
Abortion, *n* (%)		1 (3%)				1
Cesarean delivery, *n* (%)		31 (94%)	14	8	4	5
Birth weight, g	Mean	2,579	2,436	2,392	3,002	2,633
	Range	1,100–3,320	1,200–3,250	1,100–2,900	2,460–3,320	2,100–3,100
Fetal anomalies, *n* (%)		1 (3%)		1		

TM, Thalassemia major; TD-TI, transfusion–dependent Thalassemia intermedia; NTD-TI, non-transfusion-dependent Thalassemia intermedia; SCD, sickle cell disease; DFO, desferrioxamine; VOC, vaso-occlusive crisis; *mean ± standard deviation.

The thalassemic patients' group included: 14 women with thalassemia major (TM) (mean age 33.2 years, range 25–46 years) regularly transfused; 11 women with thalassemia intermedia of which 7 were transfusion dependent (mean age 34.0 years, range 25–40 years) and 4 were non-transfusion dependent (mean age 34.5 years, range 29–42 years). The SCD patients (mean age 32.1 years, range 25–43 years) were two transfusion dependent (n. 1 with double heterozygosis for HbS/HbC, and n. 1 with homozygosity HbS/HbS), and four non-transfusion dependent (n. 1 HbS/HbC and n. 3 HbS/ β-thal). All thalassemia patients were of Italian origin, while in SCD patients' group three women were of African origin, one was from Albania, and two patients had Italian origin.

### Pregnancy

In 21 pregnancies (64%) (Table 1), conception was achieved by spontaneous ovulation (seven thalassemia major, three transfusion-dependent thalassemia intermedia, five non-transfusion-dependent thalassemia intermedia, and all sickle cell disease patients), and the remaining 12 pregnancies (36%) were induced by fertilization *in vitro* in nine cases. In 3 cases, intrauterine insemination after gonadotrophin-induced ovulation was performed. The medically assisted procreations were seven thalassemia major patients affected by hypogonadotropic hypogonadism and five transfusion-dependent thalassemia intermedia. In the non-transfusion dependent thalassemia intermedia group 3 patients reported a previously pregnancy that ended with spontaneous abortion, while two have been followed for two pregnancies. Moreover, in three cases of thalassemia patients (9%) the pregnancies were twins.

### Treatment During Pregnancy

During pregnancy two cases of SCD patients (HbS/HbC) needed the transfusion of one unit of red blood cells during pregnancy for vaso-occlusive crisis (VOC), and only a case of cardiac failure occurred at 6 months of pregnancy in a thalassemia major patient with previous history of poor compliance to chelation therapy. As for iron chelation therapy during pregnancy, in the case of suspected pregnancy the chelation therapy needs to be immediately suspended, but may be resumed after the 1st trimester. In 13 thalassemic patients (10 thalassemia major and 3 transfusion-dependent thalassemia intermedia) and one SCD patient, chelation with desferrioxamine (DFO) was started again at 2nd−3rd trimester of pregnancy without complications.

### Delivery

A case of abortion for fetus death occurred in a SCD patients (HbS/HbC) after a severe vaso-occlusive crisis. This patient had already previously had a pregnancy that has resulted in abortion at 6 month due to VOC crisis and eclampsia. From the 33 pregnancies 35 children have been born, only a case of abortion (3%), with seven preterm deliveries (22%), three of which were in twin pregnancies. The cesarean delivery was applied in 31/33 pregnancies (94%), only a case of natural delivery (Table 1). The mean gestational age at delivery of all patients was 37.1 ± 2.2 weeks, with a lower mean value in TM patients (36.3 weeks) and the higher mean value in non-transfusion dependent thalassemia patients (38.4 weeks; Table 1).

### Neonatal Outcomes

The mean birth weight of the 35 children was 2,579 g, with a range from 1,100 to 3,320 g, taking into account the presence of three twin pregnancies in which the newborns weighed between 1,100 and 1,400 g and a child born at 7 month of pregnancy with birth weight of 1,700 g. These babies remained in the incubator for 15–45 days. Twenty children had breastfeeding (57%). Neonatal complications were four cases of jaundice, a case of cerebral hemorrhage, and fetal anomalies (3%) in a child of a medically assisted procreation pregnancy from ovodonation bearing gastroenteric tube multiple malformations that required several surgical interventions.

## Discussion and Conclusions

### Management of Pregnancy in Thalassemia and Sickle Cell Disease

Pregnancy of women with thalassemia major, thalassemia intermedia, and sickle cell disease should be approached by a multidisciplinary team and followed from the preconception phase until the post-partum period with a close monitoring of the maternal and fetal conditions, and planned and time delivery, in order to ensure optimal outcome. This approach requires the application of well-defined protocols that cover all the critical aspects of pregnancies in women affected by these pathologies.

Pregnancy and delivery complications are different for women with thalassemia and sickle cell disease. As regards thalassemia patients, despite the physiologic decline in fertility, and follicle aging depending on the iron toxicity, the ovarian function is preserved in transfusion-dependent thalassemia. Data on the frequency of failure in ovulation induction or timeline to a successful pregnancy are limited (Ansari et al., 2006; Voskaridou et al., 2014; Origa and Comitini, 2019). Crucial points in the management of pregnancies are the iron chelation therapy before and during pregnancy, the antithrombotic prophylaxis, the management of transfusion therapy according to the modified transfusion requirement, paying particular attention to immune-hematological examinations, and a careful cardiologic monitoring for hemodynamic changes that expose an increased risk of heart failure. The moment of the delivery must be managed in close collaboration between the Hematological Center, Obstetrics Department and Transfusion Center, taking into account the particular needs of thalassemia patients i.e., chronic anemia, cardiac siderosis, osteoporosis, splenomegaly, and patients' anatomy (fetal-pelvic discrepancy).

In sickle cell disease patients' crucial points in pregnancy management are the vaso-occlusive crisis, thromboembolic events, acute chest syndrome, anemia, and infections. Early studies recommended prophylactic transfusion during pregnancy as there was a decrease in maternal morbidity and perinatal mortality among transfused women (Malinowski et al., 2015; Sharif et al., 2018), but transfusions increase the risk of alloimmunization, iron overload and introduce the iron chelation therapy. Transfusion increases oxygen-carrying capacity and prevent complications such as stroke by correcting anemia and reducing HbS content in the circulation; therefore, a single transfusion is given. On the contrary, UK guidelines did not recommend the prophylactic transfusion during pregnancy (Sharif et al., 2018), but only in women with an history of complications such as pre-eclampsia, acute chest syndrome, stroke, and severe anemia, taking into account the previous clinical data of the sickled patients. In our SCD patients' group two cases with genotype HbS/HbC needed the transfusion of one unit of red blood cells during pregnancy for vaso-occlusive crisis.

### Iron Chelation Therapy

In the preconception period, the iron chelation therapy is strictly recommended when dealing with pregnancy forecast, in order to avoid complications due to heart diseases (heart failure and arrhythmias) or liver disease (gravid hepatosis). It is important for women to start pregnancy with low ferritin levels in order to avoid a decrease in their systolic function indices.

In the case of spontaneous pregnancy or suspected pregnancy, the chelation therapy needs to be immediately suspended, while in cases of medically assisted procreation, the iron chelation therapy should be suspended at the time of intrauterine injection of the seminal fluid or the embryonic implant, according to the involved medical procedure. Iron chelation therapy can be resumed after the first trimester of pregnancy but only with desferrioxamine (DFO), to avoid major increases in serum ferritin. DFO is the iron chelator of choice during pregnancy because its large molecular size prevents the passage through the placenta. In our patients' group, 13 thalassemia women (10 thalassemia major and 3 transfusion-dependent thalassemia intermedia) and one SCD patient started again the iron chelation therapy at 2nd−3rd trimester of pregnancy without complications, in agreement with the data reported by Tsironi et al. (2005).

### Cardiological Evaluation

Changes in cardiac function and dimension occur during pregnancy particularly in these patients (Origa et al., 2010; Lao, 2017). A close monitoring of the cardiac functions should be applied by echocardiograms and laboratory test obtained once each trimester and also after the partum in order to avoid cardiac failure in the post-partum period in women with cardiac symptoms. Myocardial complications have been reported by several authors in thalassemic pregnant women (Tuck, 2005; Ansari et al., 2006; Voskaridou et al., 2014). In our patients' group only a case of cardiac failure occurred at 6 months of pregnancy in a thalassemia major patient with previous history of poor compliance to chelation therapy.

### Immunohematological Monitoring

All transfusion-dependent patients must receive irregular antibodies search at each transfusion cycle, while non-transfusion-dependent patients search for irregular antibodies as per pregnancy calendar. It is desirable to know the extended erythrocyte phenotype of the patients' partners or of the oocyte or sperm donors in order to avoid possible alloimmunizations, where present, and possibly to prevent the onset of new alloimmunizations. The immunohematological monitoring is an essential point of the protocol of our Thalassemia Unit.

### Transfusions and Antithrombotic Prophylaxis

Anemia is common during pregnancy, and transfusion dependent patients need transfusion regime adopted on the basis of individual needs in relation to hemoglobin values which should be maintained at hemoglobin level 10 g/dL to optimize fetal growth. In addition, non-transfusion dependent thalassemia intermedia patients can need transfusion for the first time in pregnancy due to the dilution of the hemoglobin. In some cases, it may be appropriate to reduce the transfusion interval and to administer only one unit of red blood cells at a time to support the fetal oxygenation requirements (Origa and Comitini, 2019). Two cases of SCD patients (HbS/HbC) needed the transfusion of one unit of red blood cells during pregnancy for vaso-occlusive crisis.

All splenectomised patients undergo antithrombotic prophylaxis with cardioaspirin or low molecular weight heparin (LMWH), and in these cases LMWH is administered from the beginning of pregnancy. The cardioaspirin prophylaxis is replaced with LMWH at the 32nd week. Thromboprophylaxis with LMWH is be also indicated during the post-partum period to avoid thromboembolism.

### Delivery and Neonatal Outcomes

Reported rates of cesarean delivery are high, varying between 24 and 100%. The indications for this delivery are high rate of feto-pelvic disproportion and maternal short stature, osteopenia and osteoporosis, and maternal HIV infection (Leung and Lao, 2012). In our patients' group, the cesarean delivery was applied in 31/33 pregnancies (94%), only a case of natural delivery (9%) and a case of abortion for fetus death after a severe vaso-occlusive crisis occurred in a SCD patients.

Careful evaluation of obstetrics for possible fetal-pelvic discrepancy that can expose patients to pathological fractures, given the frequency of small constitution of thalassemia patients, the frequent osteopenia/osteoporosis even in young women. This evaluation is also aimed at giving an indication to cesarean delivery due to the presence of siderosis, chronic anemia, pre-existing cardiac pathologies. As a consequence, in coordination with the obstetric section and the transfusion center, blood components dedicated to transfused patients should be prepared especially in presence of specific blood components needs. Chronic anemia in these pregnant women may result in fetal hypoxia, which predisposes to premature labor, intrauterine growth retardation, and death.

In our study the observed incidence of complications was smaller compared to similar studies (Toumba et al., 2008; Kuo and Caughey, 2016; Lao, 2017; Jain et al., 2019; Origa and Comitini, 2019). Thirty two pregnancies (97%) resulted in successful deliveries, only a case of abortion (3%) in a SCD patient, no abortion in thalassemic women, only seven preterm deliveries (21%), of which three were in twin pregnancy, and severe neonatal complications only in 5.7% of children.

In conclusion, this study confirms that the progress in the multidisciplinary management of the women affected by thalassemia and sickle cell disease from the preconception phase until neonatal period, with a close monitoring of the maternal and fetal conditions. The application of well-defined protocols have been and will be in perspective the way to increase for the future the rates of pregnancies, improve the maternal and fetal outcomes, and reduce complications.

## Data Availability Statement

The raw data supporting the conclusions of this article will be made available by the authors, without undue reservation, to any qualified researcher.

## Ethics Statement

The study was approved by Comitato Etico of the S. Eugenio Hospital and the patients provided their written informed consent to participate in this study.

## Author Contributions

FS, LM, RC and DD'A enrolled the patients, performed the clinical evaluation, and management of subjects. FS, PC, RR, and SM wrote the manuscript and evaluated data for statistics. All the authors have revised and approved the final version of the manuscript.

### Conflict of Interest

The authors declare that the research was conducted in the absence of any commercial or financial relationships that could be construed as a potential conflict of interest.
